# A Light-Steered Self-Rowing Liquid Crystal Elastomer-Based Boat

**DOI:** 10.3390/polym17060711

**Published:** 2025-03-07

**Authors:** Zongsong Yuan, Jinze Zha, Junxiu Liu

**Affiliations:** 1 College of Civil Engineering, Anhui Jianzhu University, Hefei 230601, China; ys@stu.ahjzu.edu.cn (Z.Y.); zhajinze@stu.ahjzu.edu.cn (J.Z.); 2 Anhui Provincial Key Laboratory of Intelligent Geotechnics and Disaster Prevention, Anhui Jianzhu University, Hefei 230601, China

**Keywords:** self-excited motion, liquid crystal elastomer, boat, self-rowing, photothermally responsive

## Abstract

Conventional machines often face limitations due to complex controllers and bulky power supplies, which can hinder their reliability and operability. In contrast, self-excited movements can harness energy from a stable environment for self-regulation. In this study, we present a novel model of a self-rowing boat inspired by paddle boats. This boat is powered by a liquid crystal elastomer (LCE) turntable that acts as a motor and operates under consistent illumination. We investigated the dynamic behavior of the self-rowing boat under uniform illumination by integrating the photothermal reaction theory of LCEs with a nonlinear dynamic framework. The primary equations were solved using the fourth-order Runge–Kutta method. Our findings reveal that the model exhibits two modes of motion under steady illumination: a static pattern and a self-rowing pattern. The transition between these modes is influenced by the interaction of the driving and friction torques generated by photothermal energy. This study quantitatively analyzes the fundamental conditions necessary for initiating a self-rowing motion and examines how various dimensionless parameters affect the speed of the self-rowing system. The proposed system offers several unique advantages, including a simple structure, easy control, and independence from electronic components. Furthermore, it has the potential for miniaturization and integration, enhancing its applicability in miniature machines and systems.

## 1. Introduction

Self-excited motion refers to a behavior where a system shows repetitive movement in response to a sustained external stimulus [1,2,3]. A key feature of such systems is that both the extent and the length of their self-excited motion are significantly influenced by their internal characteristics, making the system highly robust [4]. Additionally, because the system can actively draw energy from a consistent external environment to sustain its periodic motion [5,6], it significantly lessens the requirement for a sophisticated control system. The self-excited motion inherently operates passively and can function autonomously without the need for external control. This characteristic enables a more rational design of systems, promoting advancements in intelligence, automation, and resource efficiency, which, in turn, enhances the overall effectiveness of the system [7,8]. The ubiquitous nature of self-excited motion has led to a wide range of applications in energy harvesting [9,10], power generation [11,12], sensing [13], soft robotics [14,15], and micro-nano devices [16,17].

Recently, there has been a notable surge in the quantity of research articles focusing on self-excited motion systems for active materials. The materials mentioned consist of hydrogels [18,19], dielectric elastomers, ionogels [20], liquid crystal elastomers (LCEs) [21,22,23], and temperature-sensitive polymers [24,25,26]. Researchers have proposed and developed various self-generated movement methods utilizing these active chemicals. These modes encompass various movements such as bending [24,25], twisting [26,27], stretching and contracting [28,29,30], rolling [31,32,33,34,35], oscillating [36,37,38,39], jumping [39,40], swimming [41], spinning [42,43,44,45], chaotic [46,47,48], self-operating device [49,50,51], and even synchronized motions in numerous interconnected self-excited resonators [52,53,54]. To ensure continuous movement in a self-excited system, it is frequently required to employ nonlinear feedback mechanisms to counteract the energy dissipation caused by damping effects in the system [55,56]. To accomplish energy replenishment, one can use various methods such as self-shadowing [31], linked motions with significant deformations [20], and combined motions involving air expansion and liquid column formation [57,58].

LCE denotes a photothermally responsive material consisting of rod-like meso-crystalline monomers [59]. These monomers feature flexible cross-linked polymers as either their main or side chains, which enable the integration of rubber-like elasticity with liquid crystal anisotropy. When exposed to external stimuli such as electricity, heat, light, or magnetism, the liquid crystal monomer molecules exhibit rotational behavior or phase changes [60,61,62]. This causes a noticeable change in their overall structure, leading to macroscopic deformation [63,64,65]. In comparison to other active materials like polyelectrolyte gels, pneumatic artificial muscles, moisture-sensitive gels, and temperature-sensitive gels, LCE is distinguished by its ability to generate self-sustained motion with enhanced responsiveness and controllability. Systems utilizing LCE for self-excited motion are known for their high reliability and consistency. This is because the LCE material has good properties that make propulsion and regulation wireless and non-contact [66,67]. These systems have a wide range of applications, showing significant potential in areas such as energy regulation [68,69], autonomous robotics [70,71], medical devices, and micro- and nano-devices [16,17].

Some recent research advances involve the development of self-excited systems using LCE materials. These systems are commonly operated by direct ambient heating or by harnessing photothermal and photochemical processes [72]. The application of 3D printing technology has particularly enhanced the performance of LCE-based robots. These innovations enable precise control of LCE material deformation in response to external stimuli without the need for complex control systems or large power supplies [73,74]. However, these systems are highly sensitive to changes in the external environment and have limited adaptability in complex environments. As a result, they still face challenges in achieving autonomous continuous motion. This study proposes an innovative paddle boat that uses the LCE turntable mechanism to achieve self-rowing. The system is driven by the rotation of the LCE turntable, featuring a lightweight design and autonomous control without relying on electronic control systems. Additionally, the system can be miniaturized and integrated, further enhancing its potential for practical applications in miniature machines and systems.

The structure of this paper is as follows. Section 2 introduces a nonlinear dynamic model for the boat system under steady illumination. This model was derived from an existing photothermal LCE system model. The model is derived from an existing photothermal LCE system model. The control equations corresponding to this model are formulated. Section 3 focuses on numerical calculations to identify the system’s static pattern and self-rowing behavior, which are computed using the fourth-order Runge–Kutta method. The investigation will explore both patterns and their underlying mechanisms. In Section 4, the article will investigate the conditions necessary for the system to initiate self-rowing and the effect of various system parameters on the speed of self-rowing. In the concluding part of the article, we will briefly summarize our findings and discuss potential future perspectives.

## 2. Model and Formulation

This section initially introduces a lightweight self-rowing boat system, featuring an LCE turntable as the motor and multiple oars. Next, the control equations for the self-rowing LCE-based boat are derived from the dynamics of the LCE and rotational mechanics. Ultimately, these control equations are further analyzed through the introduction of dimensionless system parameters.

### 2.1. Dynamics of the Self-Rowing LCE-Based Boat

**Figure 1:**
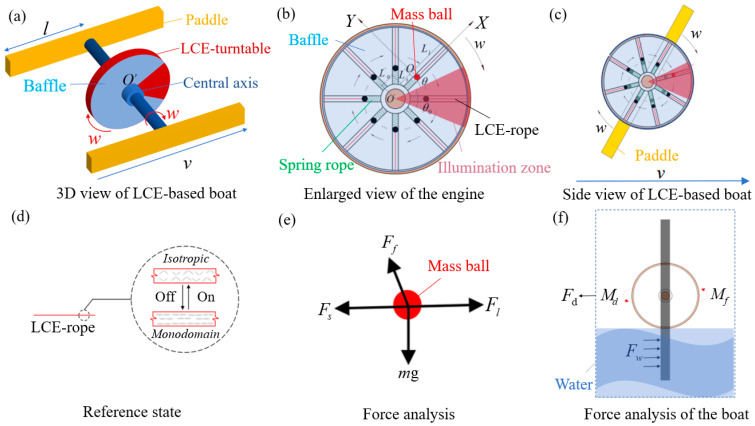
A self-rowing paddle boat system based on the LCE turntable system. (**a**) 3D view of LCE-based boat; (**b**) side view of LCE-based boat; (**c**) enlarged view of the engine; (**d**) reference state; (**e**) force analysis of the ball; (**f**) force analysis of the boat. Under steady illumination, the boat can self-rowing continuously.

A self-rowing LCE-based boat, designed based on paddle boats, has been built. It is capable of autonomously rowing in a continuous loop. Figure 1a presents a 3D view of the self-rowing LCE-based boat. It illustrates the theoretical model consisting of the LCE turntable, central axis, and several paddles. The paddles have a length of l, and the LCE turntable mechanism revolves around a point O′. The detailed diagram of the LCE turntable is shown in Figure 1b. The turntable is equipped with n motion tubes, each carrying a small mass ball, a spring of initial length $L_{s}$, and an LCE rope of initial length $L_{i}$. The LCE rope is placed at the center of the tubes, while a mass ball is positioned centrally within them. The inner side of the mass ball is attached to the turntable through a spring, and the outer side is connected to the turntable via the LCE rope. The mass ball starts at a distance of $L_{0}$ units from the center of the tube. For the light source setup, a parallel beam is used to vertically illuminate the wheel. Additionally, a baffle is fixed to the axle, ensuring that the areas of light and shadow remain constant throughout the boat’s self-rowing process. The illuminated zone is defined as the red region positioned at angle $\theta_{0}$. At the initial position, the first mass ball forms an angle of θ with the upper edge of the illuminated zone. A right-angle coordinate system Oxy is established with the initial position of the first mass ball as the origin O. As shown in Figure 1c, the turntable is set into motion with a clockwise angular velocity $w_{0}$, driving the movement of the paddles, and the current length L(t) of the LCE rope is recorded during this motion.

Figure 1d illustrates the photothermal response of the LCE rope under steady illumination. The LCE material consists of rod-shaped, asymmetric liquid crystal molecules combined with stretchable long-chain polymers, enabling the material to achieve large, reversible driving strains. This capability is primarily facilitated by the transition from the monodomain to the isotropic phase [21]. When the LCE rope enters the illuminated zone, the photothermally responsive effect causes a phase transition in the liquid crystal layer, changing it from a monodomain state to an isotropic state, which results in the contraction of the LCE rope. When the LCE-rope exits the illuminated zone, the liquid crystal layer returns to the monodomain state, and the contraction is reversed, restoring the rope to its original length. This length is referred to as the reference state, corresponding to its natural length when not exposed to light. Notably, the triangular shape of the illuminated zone ensures more precise control of light exposure, helping maintain a consistent distribution of light intensity. This is essential for the contraction behavior of the LCE-rope, contributing to stable self-propulsion. A triangular zone also helps preserve the balance of light and shadow areas, which is crucial for the system’s effective operation. Figure 1d depicts the process’s current phase, where after the LCE rope enters the illuminated zone, the boat begins to self-row.

Figure 1e illustrates the analysis of the forces on the mass-ball, including the gravitational force *mg*, the damping force $F_{f}$, the elastic force $F_{s}$ from the spring, and the tension $F_{l}$ from the LCE rope. Under the tension of the LCE rope, the mass ball continuously enters into the illumination zone and moves outward. This movement generates a torque differential, enabling the system to sustain cyclic motion and achieve dynamic equilibrium, thus ensuring continuous and stable operation of the entire LCE motor. Some paddles are attached to the LCE motor to enable the boat to row itself. Meanwhile, as the oars are rowing, the boat experiences a driving force $F_{d}$ from the water acting on the oars, along with a damping force $F_{w}$, the driving moment $M_{d}$, the maximum friction moment $M_{f}$, and the drag moment $M_{w}$ of the water on the wheel, as shown in Figure 1f.

Based on the force analysis shown in Figure 1f, from the momentum moment theorem [75], the control equations can be derived while the boat is engaged in self-rowing:

(1) $$J\frac{d^{2}\theta (t)}{dt^{2}}=M_{d}-M_{w}-M_{f},$$

where $J=\frac{1}{2}MR^{2}$ is the moment of inertia of the LCE turntable, θ(t) is the rotational angle, $\dot{\theta }=\frac{d\theta (t)}{dt}$ denotes the rotational angular velocity of the system, and $\ddot{\theta }=\frac{d\dot{\theta }(\bar{t})}{d\bar{t}}$ denotes the rotational angular velocity of the system.

Meanwhile, for the case of steady paddling by the oars. The moment equilibrium equation can be expressed as follows:

(2) $$M_{d}=M_{w}+M_{f}.$$

For stable rowing, the driving force is balanced with the damping force, i.e.,

(3) $$F_{d}=F_{w}.$$

It is assumed that the damping force is proportional to the paddling speed of the LCE-based boat, expressed as follows:

(4) $$F_{w}=\delta \cdot v,$$

where $v=\dot{\theta }\cdot l$, represents the paddling speed of the entire LCE-based boat, and δ is the damping factor of the ship.

Combining Equations (3) and (4) above, we obtain the following:

(5) $$F_{d}=\delta \cdot v,$$

where the drag moment $M_{w}$ of the water on the wheel can be derived:

(6) $$M_{w}=F_{d}l.$$

Within the LCE turntable system, the turntable begins to rotate as a result of the thermal contraction of the LCE. According to the previous model of the thermal LCE turntable system [76], the driving torque of the LCE-based boat system can be determined as

(7) $$M_{d}=\sum_{i=1}^{n}\left\{mg\cos\left[\frac{\theta_{0}}{2}-\theta -\frac{2\pi }{n}(i-1)\right]x_{i}-\beta \dot{\theta }{\left(L_{0}+x_{i}\right)}^{2}\right\},$$

where $J_{i}=m{\left(L_{0}+x_{i}\right)}^{2}$, β is the damping factor, g is the gravitational acceleration, $x_{i}$ is the displacement of the i-th mass ball.

As shown in Figure 1b, when the motion tube enters the illumination zone, the LCE rope undergoes photothermally shrinkage, pulling the mass ball outward from the turntable. The displacement of the mass ball, denoted as x(t), creates a gravitational moment difference that facilitates the system’s rotation. The sphere of mass is subjected to the following forces within the illuminated zone: gravity mg, LCE-rope-generated tension $F_{l}$, spring force $F_{s}$, and damping force $F_{f}$. It is assumed that gravity is negligible compared to the elastic force. Since the ball is in equilibrium along the x-axis at all times, its equilibrium equation can be expressed as follows:

(8) $$F_{s}=F_{l}.$$

We can simplify equilibrium Equation (8) using Hooke’s law [77], resulting in the following:

(9) $$F_{s}=-k_{s}x(t),\;F_{l}=k_{l}[x(t)+L_{i}\varepsilon_{T}(t)],$$

where $k_{s}$ refers to the elastic stiffness of the spring, $k_{l}$ refers to the elastic stiffness of the LCE rope, and $\varepsilon_{T}(t)$ is the thermally driven contraction strain of the LCE rope.

Substituting Equation (9) into Equation (8) for calculation, we can obtain:

(10) $$x_{i}(t)=-\frac{k_{l}\varepsilon_{T}(t)L_{i}}{k_{s}+k_{l}}(i=1,2,3\ldots ,n),$$

where $x_{i}(t)$ is the displacement of the i-th mass ball.

The thermally driven contraction strain of the LCE rope can be calculated as

(11) $$\varepsilon_{T}(t)=-\alpha T,$$

where α denotes the coefficient of thermal contraction of the LCE rope, and T represents the temperature difference between the LCE rope and the environment.

So, substituting Md into the control Equation (7) gives the following:

(12) $$\ddot{\theta }=\frac{\sum_{i=1}^{n}\left\{mg\cos\left[\frac{\theta_{0}}{2}-\theta -\frac{2\pi }{n}(i-1)\right]x_{i}-\beta \dot{\theta }{\left(L_{0}+x_{i}\right)}^{2}\right\}-\delta \dot{\theta }l^{2}-M_{f}}{MR^{2}}.$$

### 2.2. Photothermally Responsive LCE Model

This section outlines the approach for determining temperature using Equations (10) and (11) through the development of a photothermally responsive LCE rope model. As the LCE-based engine rotates autonomously, it undergoes heat transfer between the LCE rope and its surrounding environment. We assume that the radius r of the LCE rope is much smaller than its length $L_{i}$, allowing us to treat the temperature along its longitudinal direction as uniform. We can obtain the temperature of the LCE rope under steady illumination by the following:

(13) $$\dot{T}=\frac{q-KT}{\rho_{c}},$$

where q is the heat flux from the steady illumination, K is the heat transfer coefficient, and $\rho_{c}$ is to the specific heat capacity.

By solving Equation (13), in the illumination zone, the temperature T of the LCE rope can be determined using the instantaneous temperature $T_{\mathrm{dark}}$ and the following equation:

(14) $$T=T_{\mathrm{dark}}+T_{0}(1-e^{-t_{1}/\tau }),$$

where $T_{0}=\frac{q}{K}$ is the limiting temperature difference in the photothermally responsive LCE rope in the illumination zone, $t_{1}$ denotes the duration of current process, and $\tau =\frac{\rho_{c}}{K}$ is the characteristic time of heat exchange between the LCE rope and the environment. The value of τ is proportional to the time required for the LCE to attain the limiting temperature difference $T_{0}$.

In the dark zone, we can assume the limiting temperature difference $T_{0}$ between the LCE rope and the environment to be zero. The temperature T of the LCE rope can be determined using the instantaneous temperature $T_{\mathrm{illum}}$ and the following equation:

(15) $$T=T_{\mathrm{illum}}e^{-t_{2}/\tau },$$

where $t_{2}$ represents the time spent in the dark zone.

### 2.3. Nondimensionalization and Solution Method

For convenience, the dimensionless quantities are introduced as follows: $\bar{x_{1}}(t)=\frac{x_{1}(t)}{L_{i}}$, $\bar{x_{2}}(t)=\frac{x_{2}(t)}{L_{i}}$, $\bar{g}=\frac{g\tau^{2}}{L_{i}}$, $\bar{\beta }=\frac{\beta \tau }{m}$, $\bar{\delta }=\frac{\delta \tau }{m}$, $\bar{\alpha }=\alpha \cdot T_{e}$ (where $T_{e}$ is the environmental temperature), $\bar{t}=\frac{t}{\tau }$, ${\bar{L}}_{0}=\frac{L_{0}}{L_{i}}$, ${\bar{L}}_{s}=\frac{L_{s}}{L_{i}}$, $\bar{l}=\frac{l}{L_{i}}$, $\bar{M}=\frac{M}{m}$, ${\bar{F}}_{l}=\frac{F_{l}\tau^{2}}{mL_{i}}$, ${\bar{F}}_{s}=\frac{F_{s}\tau^{2}}{mL_{i}}$, ${\bar{M}}_{f}=\frac{M_{f}}{mgL_{i}}$, $\bar{L}(t)=\frac{L(t)}{L_{i}}$, $\bar{T}=\frac{T}{T_{e}}$, ${\bar{T}}_{0}=\frac{T_{0}}{T_{e}}$, ${\bar{k}}_{s}=\frac{k_{s}\tau^{2}}{m}$, ${\bar{k}}_{l}=\frac{k_{l}\tau^{2}}{m}$ and $\bar{\dot{\theta }}=\frac{d\theta (\bar{t})}{d\bar{t}}$.

Substituting the dimensionless parameters into Equations (10) and (12) yields the dimensionless control equations:

(16) $${\bar{x}}_{i}(\bar{t})=\frac{{\bar{k}}_{l}\bar{\alpha }\bar{T}}{{\bar{k}}_{s}+{\bar{k}}_{l}},$$

(17) $$\bar{\ddot{\theta }}=\frac{\sum_{i=1}^{n}\left\{\bar{g}\cos\left[\frac{\theta_{0}}{2}-\theta -\frac{2\pi }{n}(i-1)\right]\frac{{\bar{k}}_{l}\bar{\alpha }\bar{T}}{{\bar{k}}_{s}+{\bar{k}}_{l}}-\bar{\beta }\bar{\dot{\theta }}{\left({\bar{L}}_{0}+\frac{{\bar{k}}_{l}\bar{\alpha }\bar{T}}{{\bar{k}}_{s}+{\bar{k}}_{l}}\right)}^{2}\right\}-\bar{\delta }\bar{\dot{\theta }}\bar{l^{2}}-{\bar{M}}_{f}}{\bar{M}{\bar{R}}^{2}},$$

(18) $$\bar{v}=\bar{\dot{\theta }}\cdot \bar{l},$$

where $\bar{\ddot{\theta }}=\frac{d^{2}\theta (\bar{t})}{d\bar{t^{2}}}$ and $\bar{\dot{\theta }}=\frac{d\theta (\bar{t})}{d\bar{t}}$ represent the dimensionless rotational angular acceleration and velocity of the system, respectively.

The initial condition of the system can be given as

(19) $$\theta =1.75\pi \;\mathrm{and}\;\bar{w}=1\mathrm{at}\;\bar{t}=0.$$

Considering the dimensionless parameters including ${\bar{M}}_{f}$, ${\bar{T}}_{0}$, $\bar{g}$, $\bar{\alpha }$, ${\bar{L}}_{0}$, $\bar{l}$, $\bar{\beta }$, $\bar{\delta }$, ${\bar{k}}_{l}$, ${\bar{k}}_{s}$, $\theta_{0}$, θ, and $\bar{w}$. In MATLAB R2016b, Equations (7), (8), (10), and (11) can be solved programmatically using the fourth-order Runge–Kutta method. In the calculation, with the position $x_{i-1}$ of the mass ball at the previous moment and the temperature change ${\bar{T}}_{i}$ of the LCE, the current shrinkage strain ${\bar{\varepsilon }}_{i}$ of the LCE can be estimated by Equation (8), which is then combined with Equation (13) to calculate the current position $x_{i}$ of the mass ball. On the basis of ${\bar{\varepsilon }}_{i}$, $x_{i}$, and Equation (17), the current rotation angular velocity of the system can be calculated. When $0\leq \mathrm{mod}(\theta_{i},2\pi )\leq \theta_{0}$, the mass ball is within the illumination zone; otherwise, it is in the dark zone. Repeating the above process, the time course of the rotation angle of the LCE−based turntable system is obtained via iterative calculation.

## 3. Two Motion Patterns and Mechanism of Self-Excited Motion

In this section, we will utilize the previously established control equations to conduct a numerical analysis and explicitly examine the system’s dynamic behavior under steady illumination. First, we will introduce the two modes of motion: the static mode and the self-rowing mode. Next, we will examine the kinematics of paddleboats powered by LCE turntable engines and analyze how the main parameters influence their behavior.

### 3.1. Two Motion Patterns

**Figure 2:**
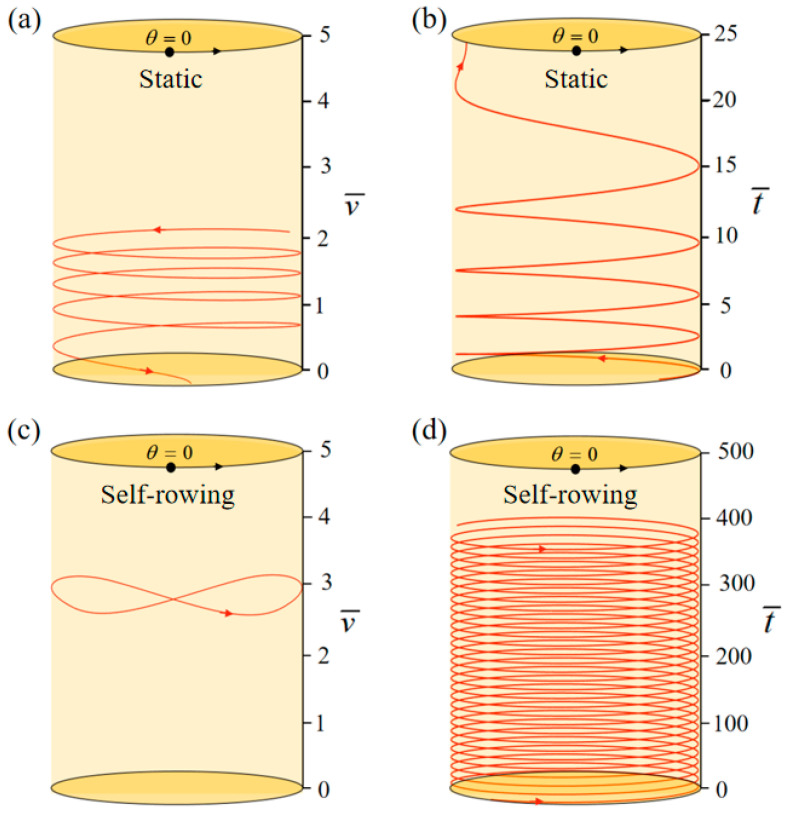
The time histories and phase trajectories of the LCE-based boat system for the two main motion modes. (**a**,**b**) Static pattern with parameters of ${\bar{M}}_{f}=0.4$, ${\bar{T}}_{0}=0$, $\bar{g}=10$, $\bar{\alpha }=0.5$, ${\bar{L}}_{0}=1$, $\bar{l}=3$, $\bar{\beta }=0.01$, $\bar{\delta }=0.01$, ${\bar{k}}_{l}=20$, ${\bar{k}}_{s}=10$, $\theta_{0}=0.5\pi$, θ=0.25π, and ${\bar{w}}_{0}=1$. (**c**,**d**) Self-rowing with parameters of ${\bar{M}}_{f}=0.4$, ${\bar{T}}_{0}=0.8$, $\bar{g}=10$, $\bar{\alpha }=0.5$, ${\bar{L}}_{0}=1$, $\bar{l}=3$, $\bar{\beta }=0.01$, $\bar{\delta }=0.01$, ${\bar{k}}_{l}=20$, ${\bar{k}}_{s}=10$, $\theta_{0}=0.5\pi$, θ=0.25π, and ${\bar{w}}_{0}=1$.

Based on existing experimental data, Table 1 and Table 2 list the system’s material properties, geometric parameters, and related dimensionless parameters [78,79,80]. An in-depth analysis requires a clear and comprehensive description of the fundamental material attributes and geometrical measurements of the system, which Table 1 offers. The appropriate dimensionless equations and the fundamental data shown in Figure 1 and Figure 2 were used to determine the associated dimensionless parameters, which are displayed in Table 2. To analyze how rotation affects the rotary table system, these characteristics are crucial. The self-rowing properties of the system under steady illumination will be assessed in this study using the values of these parameters.

The phase trajectory and time course of the system’s self-rowing under steady illumination may be determined using Equations (16) and (17). First, we set ${\bar{M}}_{f}=0.4$, ${\bar{T}}_{0}=0$, $\bar{g}=10$, $\bar{\alpha }=0.5$, ${\bar{L}}_{0}=1$, $\bar{l}=3$, $\bar{\beta }=0.01$, $\bar{\delta }=0.01$, ${\bar{k}}_{l}=20$, ${\bar{k}}_{s}=10$, $\theta_{0}=0.5\pi$, θ=0.25π, and ${\bar{w}}_{0}=1$. With these settings, the turntable rotates at a starting velocity of ${\bar{w}}_{0}=1$. Since ${\bar{T}}_{0}=0$ represents zero illumination intensity, when the LCE rope enters the illumination zone, it remains unchanged, and the turntable continues to rotate. Due to damping, the self-rowing speed of the system gradually decreases and eventually stabilizes, leading to the static mode depicted in Figure 2a,b. When we set the parameters are ${\bar{M}}_{f}=0.4$, ${\bar{T}}_{0}=0.8$, $\bar{g}=10$, $\bar{\alpha }=0.5$, ${\bar{L}}_{0}=1$, $\bar{l}=3$, $\bar{\beta }=0.01$, $\bar{\delta }=0.01$, ${\bar{k}}_{l}=20$, ${\bar{k}}_{s}=10$, $\theta_{0}=0.5\pi$, θ=0.25π, and ${\bar{w}}_{0}=1$, as shown in Figure 2c,d. The self-rowing system based on the LCE turntable may also accomplish paddling motion under steady illumination, just like other self-excited motion systems. The fundamental cause of this phenomenon is the external energy input, which cancels out the damping losses and keeps the system moving continuously. In Section 3.2, the intricate mechanism underlying this self-rowing motion is thoroughly examined.

### 3.2. Mechanism of the Self−Rowing

**Figure 3:**
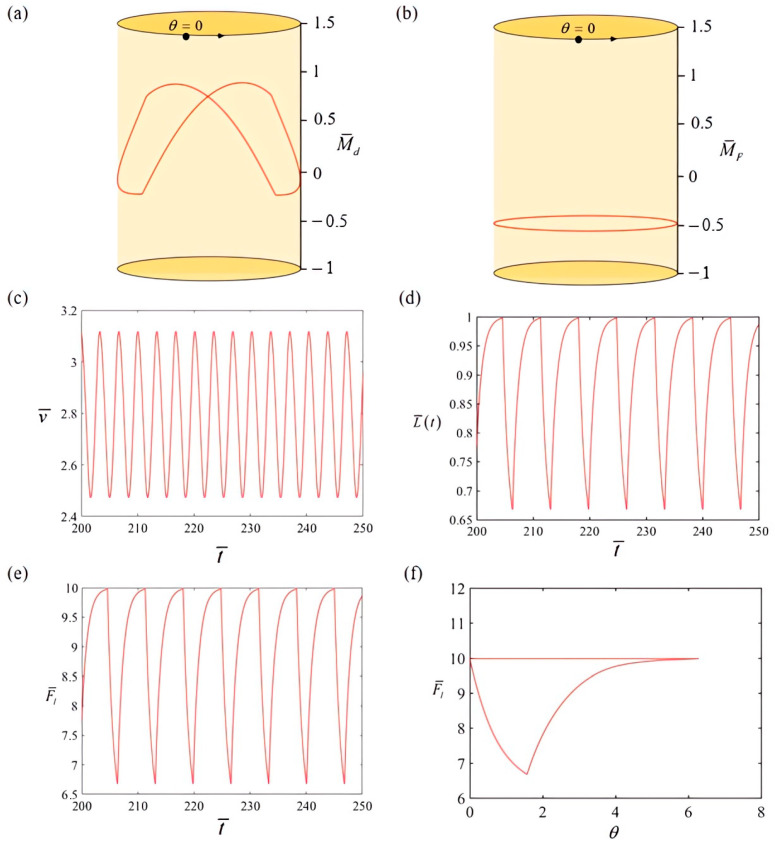
The cyclical variation in key kinematic parameters of the system in self-rowing mode. (**a**) Torque applied during self-rowing as a function of the angle of rotation; (**b**) torque from damping as a function of the angle of rotation; (**c**) boat self-rowing speed $\bar{v}$ as a function of time; (**d**) change in position of the mass sphere $\bar{L}(t)$ as a function of time; (**e**) elastic force ${\bar{F}}_{l}$ as a function of time; and (**f**) elastic force ${\bar{F}}_{l}$ as a function of angle.

Figure 3 depicts the variations in several significant characteristics linked to the rotation demonstrated in Figure 2c,d. This helps in understanding how the system achieves self-rotation. Figure 3a,b illustrates the relationship between drive torque, damping torque, and rotation angle. The area between the two lines represents the integrated effect of drive torque and damping torque, yielding a network value of 0.295. The driving torque’s positive network offsets the energy dissipated by damping, enabling the system to maintain a steady and stable rotational motion. Figure 3c,d illustrates the change over time in the boat’s self-rowing speed and the length of the LCE rope, respectively. Both numbers display cyclic fluctuations over time. Figure 3e,f shows the relationship between time and angle and the elastic force produced by the LCE rope, respectively. The periodic fluctuations in the elastic force are clearly apparent.

**Figure 4:**
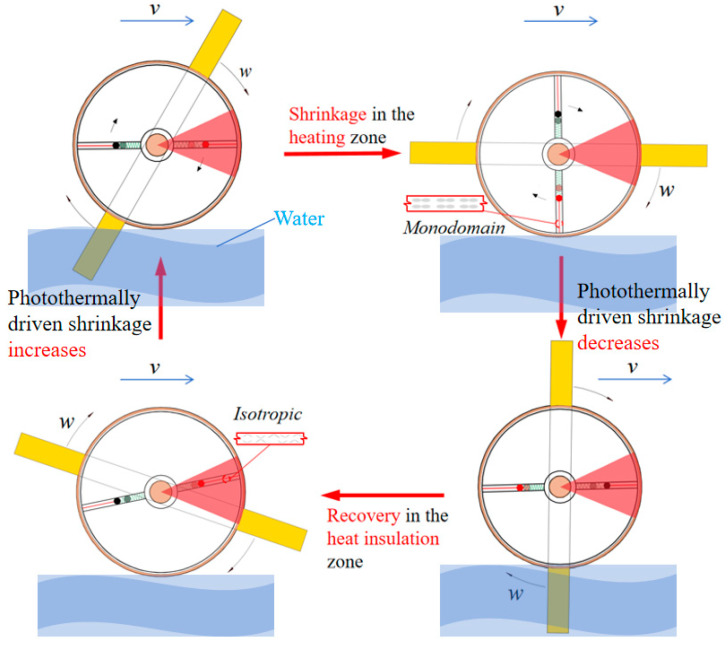
The movement of a LCE-based boat throughout a self-rowing cycle. The boat is propelled forward continuously by the shrinkage and recovery of the LCE rope, driven by the photothermal effect, completing a full self-rowing cycle.

Figure 4 illustrates the movement of an LCE-based self-rowing boat throughout a complete self-rowing cycle. Under the influence of illumination, the LCE rope shrinks, causing its length to decrease. Due to the contraction of the LCE rope, the attached mass ball shifts and moves outward. The displacement of the mass ball generates a torque, which drives the LCE turntable to rotate. The rotation of the LCE turntable, in turn, drives the paddles of the boat, creating a rowing motion that propels the boat forward. As the LCE rope continues to shrink, the boat enters the illuminated zone, where the photothermal effect intensifies, further enhancing the shrinkage of the material. This leads to a greater displacement of the mass ball, accelerating the self-rowing motion of the boat. When the boat moves into the insulation zone, the photothermal effect of the LCE rope weakens, and the material returns to a uniform state, slowing down the shrinkage. The mass ball returns to its original position, and the LCE turntable stops rotating. This continuous cycle driven by photothermal effects ensures that the boat can self-row stably and maintain its forward momentum.

## 4. Parametric Study

Equations being considered consist of eleven dimensionless parameters, namely ${\bar{M}}_{f}$, ${\bar{T}}_{0}$, $\bar{g}$, $\bar{\alpha }$, ${\bar{L}}_{0}$, $\bar{l}$, $\bar{\beta }$, $\bar{\delta }$, ${\bar{k}}_{l}$, ${\bar{k}}_{s}$, and $\theta_{0}$. Figure 2 and Figure 3 demonstrate the significant influence of these parameters on the self-rowing dynamics of the LCE-based boat system. This section of the study examines the impact of critical circumstances and motion speed on the self-rowing movement of a boat system consisting of only two mass balls in the LCE turntable system. This section accomplishes this by making adjustments to these key parameters. The objective is to offer valuable knowledge that may be applied across diverse domains, including energy harvesting, power generation, sensing, soft robotics, medical devices, and micro- and nano-devices. Here, $\bar{v}$ denotes the self-rowing speed of the boat.

### 4.1. Influence of the Maximum Frictional Torque

**Figure 5:**
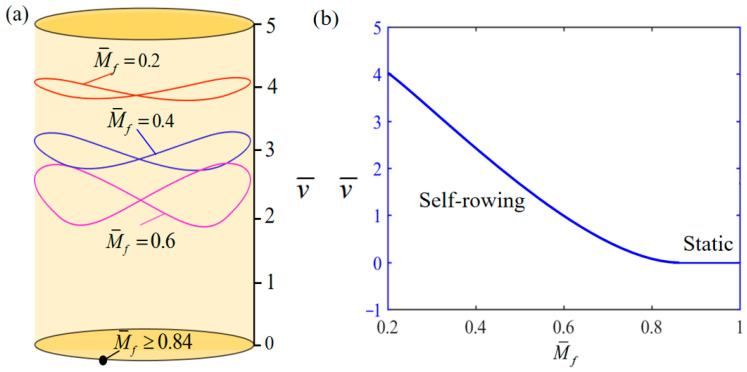
Influence of the dimensionless maximum frictional torque ${\bar{M}}_{f}$ on the self-rowing of the system, with ${\bar{T}}_{0}=0.8$, $\bar{g}=10$, $\bar{\alpha }=0.5$, ${\bar{L}}_{0}=1$, $\bar{l}=3$, $\bar{\beta }=0.01$, $\bar{\delta }=0.01$, ${\bar{k}}_{l}=20$, ${\bar{k}}_{s}=10$, and $\theta_{0}=0.5\pi$. (**a**) Limit cycles; (**b**) The self-rowing speed of a boat driven by an LCE turntable. As ${\bar{M}}_{f}$ increases, self-rowing speed $\bar{v}$ of the system’s self-rowing decreases.

Figure 5 demonstrates the influence of variation of ${\bar{M}}_{f}$ on the system self-rowing start-up conditions and speed $\bar{v}$ while parameters ${\bar{T}}_{0}=0.8$, $\bar{g}=10$, $\bar{\alpha }=0.5$, ${\bar{L}}_{0}=1$, $\bar{l}=3$, $\bar{\beta }=0.01$, $\bar{\delta }=0.01$, ${\bar{k}}_{l}=20$, ${\bar{k}}_{s}=10$, and $\theta_{0}=0.5\pi$ are held constant. Figure 5a demonstrates the relationship between the parameters and the limiting rotation period. There is a critical parameter ${\bar{M}}_{f}=0.84$, which, when ${\bar{M}}_{f}\geq 0.84$, results in the system remaining stationary. This phenomenon is attributed to the fact that when the frictional torque is too large, the energy input to the system is not sufficient to offset the energy consumed due to the damping effect. Conversely, when ${\bar{M}}_{f}=$ 0.2, 0.4, 0.6 is attained, the system enters into self-rowing motion. The variation in the self-rowing speed $\bar{v}$ with the parameter is depicted in Figure 5b, revealing that $\bar{v}$ decrease as the parameter increases. Equation (19) shows that the rotation velocity $\bar{\dot{\theta }}$ declines as ${\bar{M}}_{f}$ increases.

### 4.2. Influence of the Limit Temperature

**Figure 6:**
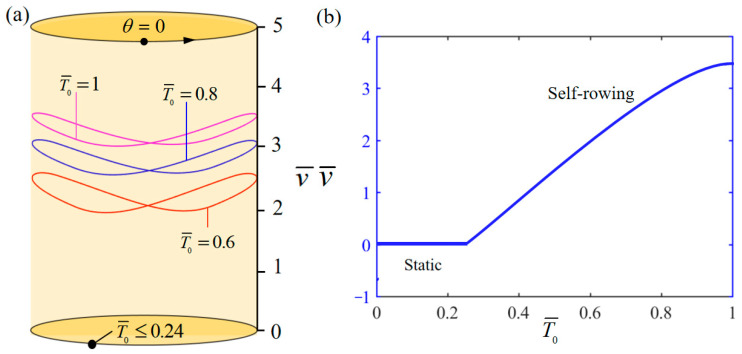
Influence of the dimensionless limit temperature ${\bar{T}}_{0}$ on the self-rowing of the system, with ${\bar{M}}_{f}=0.4$, $\bar{g}=10$, $\bar{\alpha }=0.5$, ${\bar{L}}_{0}=1$, $\bar{l}=3$, $\bar{\beta }=0.01$, $\bar{\delta }=0.01$, ${\bar{k}}_{l}=20$, ${\bar{k}}_{s}=10$, and $\theta_{0}=0.5\pi$. (**a**) Limit cycles; (**b**) the self-rowing speed of a boat driven by an LCE turntable. As ${\bar{T}}_{0}$ increases, self-rowing speed $\bar{v}$ of the system’s self-rowing increases.

Figure 6 demonstrates the influence of variation of ${\bar{T}}_{0}$ on the system self-rowing start-up conditions and speed $\bar{v}$ while parameters ${\bar{M}}_{f}=0.4$, $\bar{g}=10$, $\bar{\alpha }=0.5$, ${\bar{L}}_{0}=1$, $\bar{l}=3$, $\bar{\beta }=0.01$, $\bar{\delta }=0.01$, ${\bar{k}}_{l}=20$, ${\bar{k}}_{s}=10$, and $\theta_{0}=0.5\pi$ are held constant. Figure 6a demonstrates the relationship between the parameters and the limiting rotation period. There is a critical parameter ${\bar{T}}_{0}=0.24$, which, when ${\bar{T}}_{0}\leq 0.24$, results in the system remaining stationary. This phenomenon is attributed to the fact that when the limiting temperature is low, the photothermal energy input to the system is not sufficient to offset the energy consumed due to the damping effect. Conversely, when ${\bar{T}}_{0}=$ 0.6, 0.8, 1 is attained, the system enters into a self-rowing motion. The variation in the self-rowing speed $\bar{v}$ with the parameter is depicted in Figure 6b, revealing that $\bar{v}$ increases as the parameter increases. This is due to the fact that as the temperature continues to rise, the mechanical energy of the system due to the conversion of photothermal energy continues to increase while the energy dissipated due to damping remains constant, resulting in an increasing speed of the system.

### 4.3. Influence of the Gravitational Acceleration

**Figure 7:**
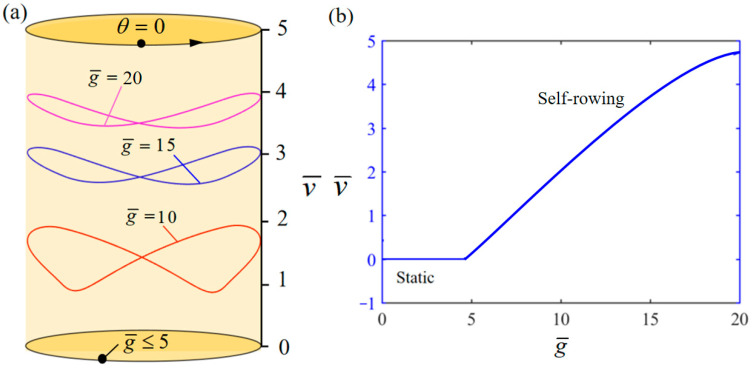
Influence of the dimensionless gravitational acceleration $\bar{g}$ on the self-rowing of the system, with ${\bar{M}}_{f}=0.4$, ${\bar{T}}_{0}=0.8$, $\bar{\alpha }=0.5$, ${\bar{L}}_{0}=1$, $\bar{l}=3$, $\bar{\beta }=0.01$, $\bar{\delta }=0.01$, ${\bar{k}}_{l}=20$, ${\bar{k}}_{s}=10$, and $\theta_{0}=0.5\pi$. (**a**) Limit cycles; (**b**) the self-rowing speed of a boat driven by an LCE turntable. As $\bar{g}$ increases, self-rowing speed $\bar{v}$ of the system’s self-rowing increases.

Figure 7 demonstrates the influence of variation of $\bar{g}$ on the system self-rowing start-up conditions and speed $\bar{v}$ while parameters ${\bar{M}}_{f}=0.4$, ${\bar{T}}_{0}=0.8$, $\bar{\alpha }=0.5$, ${\bar{L}}_{0}=1$, $\bar{l}=3$, $\bar{\beta }=0.01$, $\bar{\delta }=0.01$, ${\bar{k}}_{l}=20$, ${\bar{k}}_{s}=10$, and $\theta_{0}=0.5\pi$ are held constant. Figure 7a demonstrates the relationship between the parameters and the limiting rotation period. There is a critical parameter $\bar{g}=5$, when the $\bar{g}\leq 5$, the torque produced by the system is too small. As a result, the energy lost to damping will exceed the photothermal energy generated by the system. The self-rowing LCE-based boat system eventually ceases motion and transitions into a state of static equilibrium. Conversely, when $\bar{g}=$5, 10, 15 is attained, the system enters into a self-rowing motion. The variation in the self-rowing speed $\bar{v}$ with the parameter is depicted in Figure 7b, revealing that $\bar{v}$ increases as the parameter increases.

### 4.4. Influence of the Thermal Shrinkage Coefficient

**Figure 8:**
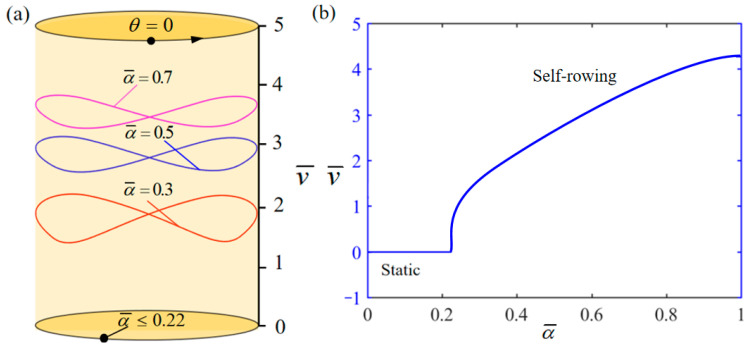
Influence of the dimensionless thermal shrinkage coefficient $\bar{\alpha }$ on the self-rowing, with ${\bar{M}}_{f}=0.4$, ${\bar{T}}_{0}=0.8$, $\bar{g}=10$, ${\bar{L}}_{0}=1$, $\bar{l}=3$, $\bar{\beta }=0.01$, $\bar{\delta }=0.01$, ${\bar{k}}_{l}=20$, ${\bar{k}}_{s}=10$, and $\theta_{0}=0.5\pi$. (**a**) Limit cycles; (**b**) the self-rowing speed of a boat driven by an LCE turntable. As $\bar{\alpha }$ increases, the self-rowing speed $\bar{v}$ of the system’s self-rowing increases.

Figure 8 demonstrates the influence of variation of $\bar{\alpha }$ on the system self-rowing start-up conditions and speed $\bar{v}$ while parameters ${\bar{M}}_{f}=0.4$, ${\bar{T}}_{0}=0.8$, $\bar{g}=10$, ${\bar{L}}_{0}=1$, $\bar{l}=3$, $\bar{\beta }=0.01$, $\bar{\delta }=0.01$, ${\bar{k}}_{l}=20$, ${\bar{k}}_{s}=10$, and $\theta_{0}=0.5\pi$ are held constant. Figure 8a demonstrates the relationship between the parameters and the limiting rotation period. There is a critical parameter $\bar{\alpha }=0.22$, which, when reached, results in the system remaining stationary. Conversely, when $\bar{\alpha }=$ 0.3, 0.5, and 0.7, is attained, the system enters into self-rowing motion. The variation in the self-rowing speed $\bar{v}$ with the parameter is depicted in Figure 8b, revealing that $\bar{v}$ increases as the parameter increases. The increase in shrinkage factor, as illustrated in Equation (11), results in a growth in the photothermally driven shrinkage strain. This leads to a corresponding rise in the absorbed photothermal energy. Thus, it can be concluded that enhancing the shrinkage coefficient of LCE materials boosts the effectiveness of converting photothermal energy into mechanical energy.

### 4.5. Influence of the Initial Position of the Mass Ball

**Figure 9:**
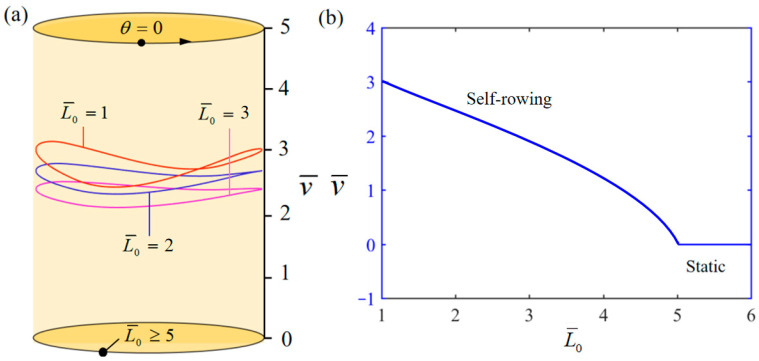
Influence of dimensionless initial position ${\bar{L}}_{0}$ on the self-rowing of the system, with ${\bar{M}}_{f}=0.4$, ${\bar{T}}_{0}=0.8$, $\bar{g}=10$, $\bar{\alpha }=0.5$, $\bar{l}=3$, $\bar{\beta }=0.01$, $\bar{\delta }=0.01$, ${\bar{k}}_{l}=20$, ${\bar{k}}_{s}=10$, and $\theta_{0}=0.5\pi$. (**a**) Limit cycles; (**b**) the self-rowing speed of a boat driven by an LCE turntable. As ${\bar{L}}_{0}$ increases, the self-rowing speed $\bar{v}$ of the system’s self-rowing decreases.

Figure 9 demonstrates the influence of variations in ${\bar{L}}_{0}$ of the mass ball on the system self-rowing start-up conditions and speed $\bar{v}$ while parameters ${\bar{M}}_{f}=0.4$, ${\bar{T}}_{0}=0.8$, $\bar{g}=10$, $\bar{\alpha }=0.5$, $\bar{l}=3$, $\bar{\beta }=0.01$, $\bar{\delta }=0.01$, ${\bar{k}}_{l}=20$, ${\bar{k}}_{s}=10$, and $\theta_{0}=0.5\pi$ are held constant. Figure 9a demonstrates the relationship between the parameters and the limiting rotation period. There is a critical parameter ${\bar{L}}_{0}=5$. When the initial position exceeds or equals the critical value, the energy input from an external source is insufficient to compensate for the damping loss. As a result, the system’s rotation speed gradually decreases until it stops at the static equilibrium position. Conversely, when ${\bar{L}}_{0}=$ 1, 2, and 3 is attained, the system enters into self-rowing motion. The variation in the self-rowing speed $\bar{v}$ with the parameter is depicted in Figure 9b, revealing that $\bar{v}$ decrease as the parameter increases. This result demonstrates that an increase in the initial position hinders the rotation of the system, leading to a gradual slowdown and eventual halt at the static equilibrium position.

### 4.6. Influence of the Length of Paddle

**Figure 10:**
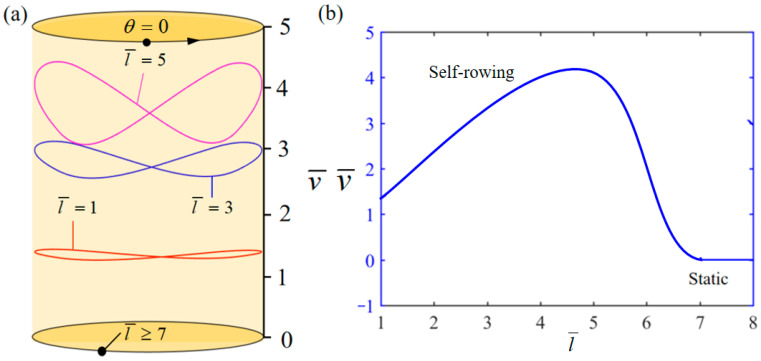
Influence of the dimensionless length $\bar{l}$ of paddle on the self-rowing of the system, with ${\bar{M}}_{f}=0.4$, ${\bar{T}}_{0}=0.8$, $\bar{g}=10$, $\bar{\alpha }=0.5$, ${\bar{L}}_{0}=1$, $\bar{\beta }=0.01$, $\bar{\delta }=0.01$, ${\bar{k}}_{l}=20$, ${\bar{k}}_{s}=10$, and $\theta_{0}=0.5\pi$. (**a**) Limit cycles; (**b**) the self-rowing speed of a boat driven by an LCE turntable. As $\bar{l}$ increases, the self-rowing speed $\bar{v}$ of the system’s self-rowing increases and then decreases.

Figure 10 demonstrates the influence of variations in the length $\bar{l}$ of paddle on the system self-rowing start-up conditions and speed $\bar{v}$ while parameters ${\bar{M}}_{f}=0.4$, ${\bar{T}}_{0}=0.8$, $\bar{g}=10$, $\bar{\alpha }=0.5$, ${\bar{L}}_{0}=1$, $\bar{\beta }=0.01$, $\bar{\delta }=0.01$, ${\bar{k}}_{l}=20$, ${\bar{k}}_{s}=10$, and $\theta_{0}=0.5\pi$ are held constant. Figure 10a demonstrates the relationship between the parameters and the limiting rotation period. There is a critical parameter $\bar{l}=7$, which, when $\bar{l}\geq 7$, results in the system remaining stationary; the reason for this phenomenon is derived from Equation (19). As $\bar{l}$ increases, the energy consumed by the system due to damping increases. When $\bar{l}$ is too large, the energy input to the system is not enough to offset the energy consumed by the damping effect and the system will remain stationary. Conversely, when $\bar{l}=$ 1, 3, and 5 is attained, the system enters into self-rowing motion. Figure 10b illustrates the relationship between self-rowing speed and the parameters. It demonstrates a pattern of initially increasing and then decreasing as the parameters grow. Based on Equation (19), it is evident that the acceleration of the turntable rotation decreases as $\bar{l}$ increases. Hence, continuously raising the value of $\bar{l}$ does not contribute to the enhancement of the system speed.

### 4.7. Influence of the Damping Factor

**Figure 11:**
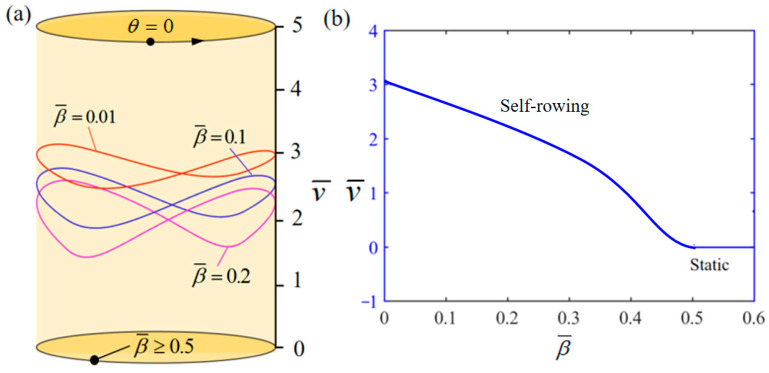
Influence of the dimensionless damping factor $\bar{\beta }$ on the self-rowing of the system, with ${\bar{M}}_{f}=0.4$, ${\bar{T}}_{0}=0.8$, $\bar{g}=10$, $\bar{\alpha }=0.5$, ${\bar{L}}_{0}=1$, $\bar{l}=3$, $\bar{\delta }=0.01$, ${\bar{k}}_{l}=20$, ${\bar{k}}_{s}=10$, and $\theta_{0}=0.5\pi$. (**a**) Limit cycles; (**b**) the self-rowing speed of a boat driven by an LCE turntable. As $\bar{\beta }$ increases, the self-rowing speed $\bar{v}$ of the system’s self-rowing decreases.

Figure 11 demonstrates the influence of variations in $\bar{\beta }$ on the system self-rowing start-up conditions and speed $\bar{v}$ while parameters ${\bar{M}}_{f}=0.4$, ${\bar{T}}_{0}=0.8$, $\bar{g}=10$, $\bar{\alpha }=0.5$, ${\bar{L}}_{0}=1$, $\bar{l}=3$, $\bar{\delta }=0.01$, ${\bar{k}}_{l}=20$, ${\bar{k}}_{s}=10$, and $\theta_{0}=0.5\pi$ are held constant. Figure 11a demonstrates the relationship between the parameters and the limiting rotation period. There is a critical parameter $\bar{\beta }=0.5$, which once the critical value is exceeded, the system’s damping dissipation becomes too significant to be offset by the mechanical energy produced by the input thermal energy. Conversely, when $\bar{\beta }=$ 0.01, 0.1, 0.2 is attained, the system enters into self-rowing motion. The variation in the self-rowing speed $\bar{v}$, with the parameter is depicted in Figure 11b, revealing that $\bar{v}$ decrease as the parameter increases. The phenomena can be attributed to the direct impact of the damping coefficient’s magnitude on energy dissipation, leading to a decrease in the amount of accessible rotational energy.

### 4.8. Influence of the Rolling Resistance Coefficient

**Figure 12:**
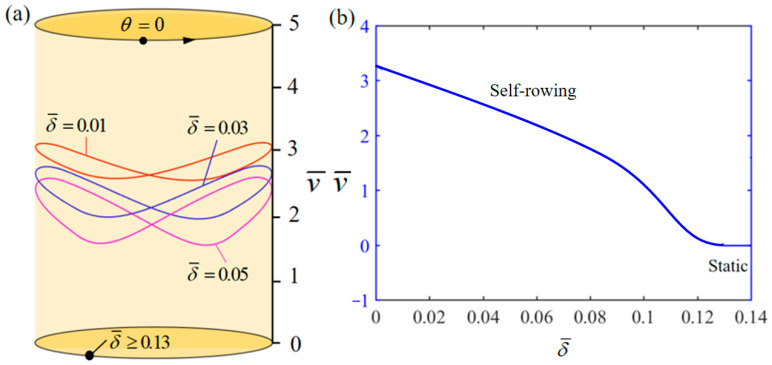
Influence of the dimensionless rolling resistance coefficient $\bar{\delta }$ on the self-rowing of the system, with ${\bar{M}}_{f}=0.4$, ${\bar{T}}_{0}=0.8$, $\bar{g}=10$, $\bar{\alpha }=0.5$, ${\bar{L}}_{0}=1$, $\bar{l}=3$, $\bar{\beta }=0.01$, ${\bar{k}}_{l}=20$, ${\bar{k}}_{s}=10$, and $\theta_{0}=0.5\pi$. (**a**) Limit cycles; (**b**) the self-rowing speed of a boat driven by an LCE turntable. As $\bar{\delta }$ increases, the self-rowing speed $\bar{v}$ of the system’s self-rowing decreases.

Figure 12 demonstrates the influence of variation of $\bar{\delta }$ on the system self-rowing start-up conditions and speed $\bar{v}$ while parameters ${\bar{M}}_{f}=0.4$, ${\bar{T}}_{0}=0.8$, $\bar{g}=10$, $\bar{\alpha }=0.5$, ${\bar{L}}_{0}=1$, $\bar{l}=3$, $\bar{\beta }=0.01$, ${\bar{k}}_{l}=20$, ${\bar{k}}_{s}=10$, and $\theta_{0}=0.5\pi$ are held constant. Figure 12a demonstrates the relationship between the parameters and the limiting rotation period. There is a critical parameter $\bar{\delta }=0.13$, which, when $\bar{\delta }\geq 0.13$, results in the system remaining stationary. Conversely, when $\bar{\delta }=$ 0.01, 0.1, and 0.2 is attained, the system enters into self-rowing motion. The variation in the self-rowing speed $\bar{v}$ with the parameter is depicted in Figure 12b, revealing that $\bar{v}$ decrease as the parameter increases. The effect of the rolling resistance coefficient on the system is similar to that of the damping coefficient, and the magnitude of both of them can have a direct effect on energy dissipation, resulting in a reduction in the available rotational energy.

### 4.9. Influence of the Elastic Stiffness of LCE−Rope

**Figure 13:**
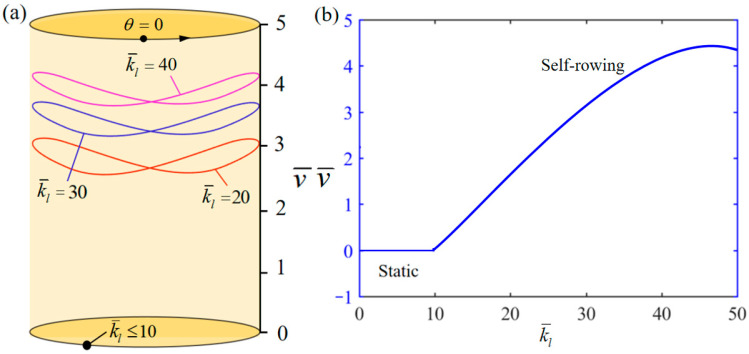
Influence of the dimensionless elastic stiffness of LCE−rope ${\bar{k}}_{l}$ on the self-rowing of the system, with ${\bar{M}}_{f}=0.4$, ${\bar{T}}_{0}=0.8$, $\bar{g}=10$, $\bar{\alpha }=0.5$, ${\bar{L}}_{0}=1$, $\bar{l}=3$, $\bar{\beta }=0.01$, $\bar{\delta }=0.01$, ${\bar{k}}_{s}=10$, and $\theta_{0}=0.5\pi$. (**a**) Limit cycles; (**b**) the self-rowing speed of a boat driven by an LCE turntable. As ${\bar{k}}_{l}$ increases, self-rowing speed $\bar{v}$ of the system’s self-rowing increases.

Figure 13 demonstrates the influence of variation of ${\bar{k}}_{l}$ on the system self-rowing start-up conditions and speed $\bar{v}$ while parameters ${\bar{M}}_{f}=0.4$, ${\bar{T}}_{0}=0.8$, $\bar{g}=10$, $\bar{\alpha }=0.5$, ${\bar{L}}_{0}=1$, $\bar{l}=3$, $\bar{\beta }=0.01$, $\bar{\delta }=0.01$, ${\bar{k}}_{s}=10$, and $\theta_{0}=0.5\pi$ are held constant. Figure 13a demonstrates the relationship between the parameters and the limiting rotation period. There is a critical parameter ${\bar{k}}_{l}=10$, which, when ${\bar{k}}_{l}\leq 10$, the elastic force ${\bar{F}}_{l}$ generated by the LCE rope is small relative to the elastic force ${\bar{F}}_{s}$ generated by the spring, and the displacement of the mass ball is small. Thus, it is not possible to generate enough gravitational momentum to maintain the continuous motion of the turntable system, which ultimately leads to the cessation of the system’s motion. Conversely, when ${\bar{k}}_{l}=$ 20, 30, and 40 is attained, the system enters into self-rowing motion. Figure 13b shows the variation in the self-rowing speed $\bar{v}$ with respect to the parameter, indicating that as the parameter increases, the self-rowing speed first increases and then remains stable. This phenomenon illustrates the decreasing effect on the system as ${\bar{k}}_{l}$ increases.

### 4.10. Influence of the Elastic Stiffness of Spring

**Figure 14:**
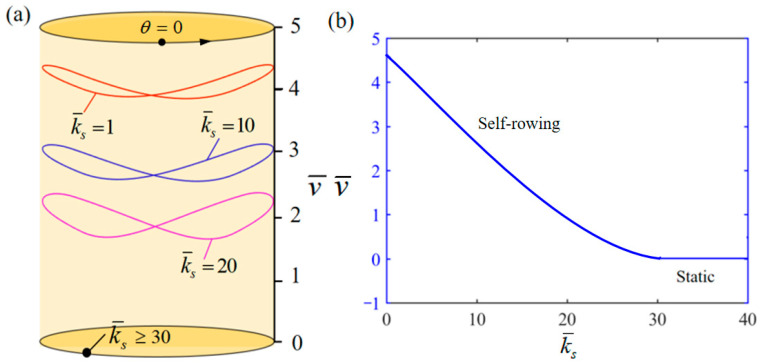
Influence of the dimensionless elastic stiffness of spring ${\bar{k}}_{s}$ on the self-rowing of the system, with ${\bar{M}}_{f}=0.4$, ${\bar{T}}_{0}=0.8$, $\bar{g}=10$, $\bar{\alpha }=0.5$, ${\bar{L}}_{0}=1$, $\bar{l}=3$, $\bar{\beta }=0.01$, $\bar{\delta }=0.01$, ${\bar{k}}_{l}=20$, and $\theta_{0}=0.5\pi$. (**a**) Limit cycles; (**b**) the self-rowing speed of a boat driven by an LCE turntable. As ${\bar{k}}_{s}$ increases, the self-rowing speed $\bar{v}$ of the system’s self-rowing decreases.

Figure 14 demonstrates the influence of variation of ${\bar{k}}_{s}$ on the system self-rowing start-up conditions and speed $\bar{v}$ while parameters ${\bar{M}}_{f}=0.4$, ${\bar{T}}_{0}=0.8$, $\bar{g}=10$, $\bar{\alpha }=0.5$, ${\bar{L}}_{0}=1$, $\bar{l}=3$, $\bar{\beta }=0.01$, $\bar{\delta }=0.01$, ${\bar{k}}_{l}=20$, and $\theta_{0}=0.5\pi$ are held constant. Figure 14a demonstrates the relationship between the parameters and the limiting rotation period. There is a critical parameter ${\bar{k}}_{s}=30$, which, when ${\bar{k}}_{s}\geq 30$, results in the system remaining stationary. Conversely, when ${\bar{k}}_{s}=$ 1, 10, and 20 is attained, the system enters into self-rowing motion. The variation in the self-rowing speed $\bar{v}$ with the parameter is depicted in Figure 14b, revealing that $\bar{v}$ decreases as the parameter increases. The reasons for this phenomenon are similar to those of ${\bar{k}}_{l}$. As ${\bar{k}}_{s}$ continues to increase, the elastic force ${\bar{F}}_{s}$ generated by the spring also increases, resulting in a gradual decrease in the displacement of the mass ball. The driving torque generated by the system will decrease, resulting in a gradual decrease in the speed of the system and finally entering a static state.

### 4.11. Influence of the Illumination Zone Angle

**Figure 15:**
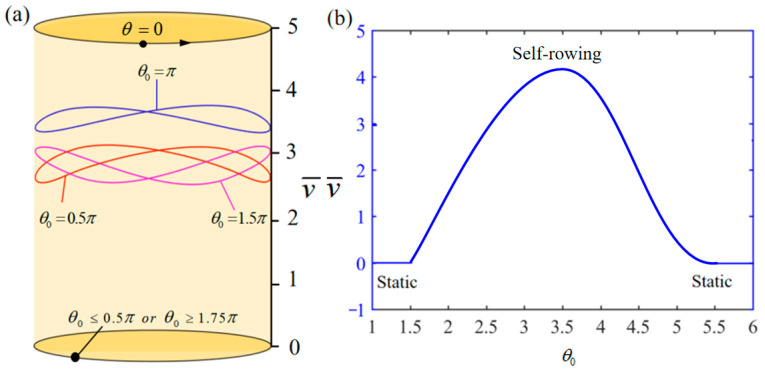
Influence of $\theta_{0}$ on the self-rowing of the system, with ${\bar{M}}_{f}=0.4$, ${\bar{T}}_{0}=0.8$, $\bar{g}=10$, $\bar{\alpha }=0.5$, ${\bar{L}}_{0}=1$, $\bar{l}=3$, $\bar{\beta }=0.01$, $\bar{\delta }=0.01$, ${\bar{k}}_{l}=20$, and ${\bar{k}}_{s}=10$. (**a**) Limit cycles; (**b**) the self-rowing speed of a boat driven by an LCE turntable. As $\theta_{0}$ increases, the self-rowing speed $\bar{v}$ of the system’s self-rowing increases and then decreases.

Figure 15 demonstrates the influence of variation in the illumination zone angle $\theta_{0}$ on the system self-rowing start-up conditions and speed $\bar{v}$ while parameters ${\bar{M}}_{f}=0.4$, ${\bar{T}}_{0}=0.8$, $\bar{g}=10$, $\bar{\alpha }=0.5$, ${\bar{L}}_{0}=1$, $\bar{l}=3$, $\bar{\beta }=0.01$, $\bar{\delta }=0.01$, ${\bar{k}}_{l}=20$, and ${\bar{k}}_{s}=10$ are held constant. Figure 15a demonstrates the relationship between the parameters and the limiting rotation period. There is a critical parameter $\theta_{0}=0.5\pi$ and $\theta_{0}=1.75\pi$, if the angle of the illumination zone is below the critical threshold, the LCE does not spend enough time in the illumination zone. As a result, the thermal energy provided is inadequate to compensate for the energy lost due to damping, which keeps the system in a state of static equilibrium. On the other hand, if the angle of the illumination zone exceeds a certain limit, the LCE rope does not remain in the dark zone long enough to revert to its original condition. As a result, the supplied thermal energy fails to offset the energy loss, resulting in the system staying in a state of static. Figure 15b demonstrates the impact of the illumination zone angle on the speed of self-rowing. The graphic illustrates a trend where the self-rowing speed initially rises and subsequently declines as the illumination zone angle increases. This is analogous to the aforementioned explanation that an excessively small or too large illumination zone range can have an adverse impact on the rotation of the system.

This section systematically examines how different key dimensionless parameters impact the self-rowing speed of the LCE-based boat, as detailed in Table 3. The provided data offer valuable insights for the engineering design of self-excited motion systems, facilitating precise control over their motion characteristics in practice. In summary, the analysis presented in Table 3 serves as a critical reference for optimizing the engineering design and operational performance of self-rowing LCE-based boats.

## 5. Conclusions

Conventional active material systems are constrained by complex controllers and bulky power supplies, reducing reliability and operability. Self-excited movements can harness energy from a stable environment and utilize it for self-regulation. This paper explores a paddle boat system capable of self-rowing under steady illumination. It proposes a nonlinear theoretical model combined with an established photothermally LCE model to investigate the dynamic behavior of the self-rowing paddle boat under steady illumination using the fourth-order Runge–Kutta method. The numerical calculations reveal that the paddle boat system exhibits two distinct modes of motion under steady illumination: static mode and self-rowing mode. It also emphasizes the indispensable correlation between photothermal energy input and damping dissipation to sustain the contact cycle motion of the system.

Additionally, the study provides an analysis of the essential conditions required to initiate self-rowing and explores how key dimensionless parameters affect the self-rowing motion of the system. The self-rowing speed of the system is determined by key parameters such as frictional moment, gravitational acceleration, paddle length, initial position of the mass ball, elastic properties of the LCE rope and springs, limiting temperature, angular range of the illumination zone, thermal contraction coefficients, damping coefficients, and roll damping coefficients. These results in this paper have important applications through further experimental validation to confirm the self-rowing phenomenon, verify the accuracy of the numerical calculations, and explore the practical applications of the phenomenon. Moreover, these findings offer new design insights for self-rowing systems, advancing our understanding of the self-rowing principle and broadening its potential applications in fields, such as renewable energy systems, environmental monitoring, advanced robotics, healthcare technologies, and micro-electromechanical systems. Self-rowing systems naturally benefit from a simple structure and sustainability, which enhances their applicability and effectiveness across diverse fields. These insights are essential for directing research and development in related domains, fostering technological breakthroughs, and enabling effective real-world applications.

## Figures and Tables

**Table 1:** Material properties and geometric parameters.

Parameter	Definition	Value	Unit
α	Thermal shrinkage coefficient of the LCE material [21,78]	0–0.5	/
g	Gravitational acceleration	10	${m/s}^{2}$
β	Damping factor	0.001~0.01	kg/s
$\theta_{0}$	illumination zone angle	0.4*π*~0.8*π*	/
θ	Initial angle of the mass ball	0~2*π*	/
$w_{0}$	Initial angular velocity	0.4~2	rad/s
$k_{s}$	Elastic stiffness of the spring	0.005~50	N/m
$k_{l}$	Elastic stiffness of the LCE rope	0.005~50	N/m
$L_{0}$	Distance from mass ball to turntable center	0.04~0.16	m
$L_{s}$	Initial length of spring	1.6~20	mm
τ	Thermal characteristic time [79]	0.001~0.1	s
$T_{0}$	Limit temperature difference in LCE-rope	0–20	°C
$\rho_{c}$	Specific heat capacity of LCE material	1000~4500	J/(kg°C)
q	Heat flux [79]	0~0.02	J/s
K	Heat transfer coefficient [80]	1	${W/m}^{2}\cdot K$

**Table 2:** Dimensionless parameters.

**Parameter**	${\bar{M}}_{f}$	${\bar{T}}_{0}$	$\bar{g}$	$\bar{\alpha }$	${\bar{L}}_{0}$	$\bar{l}$	$\bar{\beta }$	$\bar{\delta }$	${\bar{k}}_{l}$	${\bar{k}}_{s}$	$\theta_{0}$
**Value**	0–0.8	0.24–1	5–20	0.22–0.8	1–5	1–7	0–0.5	0–0.13	10–50	10–40	0.5π

**Table 3:** Influences of several key dimensionless parameters.

Parameter	$\mathrm{Self}\text{-}\mathrm{Rowing}\;\mathrm{Speed}\;\bar{v}$
${\bar{M}}_{f}$	decreases with increasing ${\bar{M}}_{f}$
$\bar{g}$	increases with increasing $\bar{g}$
${\bar{T}}_{0}$	increases with increasing ${\bar{T}}_{0}$
$\bar{\alpha }$	increases with increasing $\bar{\alpha }$
$\bar{l}$	first increases and then decreases with increasing $\bar{l}$
${\bar{L}}_{0}$	decreases with increasing ${\bar{L}}_{0}$
$\bar{\beta }$	decreases with increasing $\bar{\beta }$
$\bar{\delta }$	decreases with increasing $\bar{\delta }$
${\bar{k}}_{l}$	increases with increasing ${\bar{k}}_{l}$
${\bar{k}}_{s}$	decreases with increasing ${\bar{k}}_{s}$
$\theta_{0}$	first increases and then decreases with increasing $\theta_{0}$

## Data Availability

The original contributions presented in this study are included in the article. Further inquiries can be directed to the corresponding author.
